# Trajectories of K-12 Schools and Demographic Differences: Evidence From 2017 Life History Mail Survey

**DOI:** 10.1093/geroni/igab046.3304

**Published:** 2021-12-17

**Authors:** Wenshan Yu, Xuefei Li, Jacqui Smith

**Affiliations:** 1 Institute for Social Research, University of Michigan (Ann Arbor), Ann Arbor, Michigan, United States; 2 Institute for Social Research, University of Michigan, Ann Arbor, Michigan, United States; 3 University of Michigan, University of Michigan, Michigan, United States

## Abstract

Besides information about the highest degree, little information about early-life education is available in most population surveys. This study identified the trajectories of K-12 education history among older adults in the Health and Retirement Study born between 1930 and 1960, and examined the associations with demographic variables. Drawing on 2017 Spring and Fall Life History Mail Survey (LHMS; n = 3,206), we used sequence analysis to determine and classify trajectories of school types across the education history. We identified five trajectories: 1) always private school with White students, 2) always public school with White students, 3) always public school with Non-White students, 4) mostly private school with Non-White students, and 5) no report of school types. The trajectories showed that changes in school type (i.e. private to public) often happened in grade 9. Changes rarely happened across race/ethnicity groups (i.e. mostly White to mostly non-White). We used multinomial logistic regression to examine the relationship between demographic variables and education trajectories. We found that compared to Black participants, White participants were significantly less likely to be in mostly Non-White schools (public and private schools, p<0.001). The 1940s and 1950s cohort were more likely to join mostly White private schools than the 1930s cohort (odds ratio: 1.70 for 1940s and 1.62 for 1950s separately, p<0.005). Our findings illustrate a novel application of sequence analysis with life history data, as well as new evidence on recial segregation in early-life education within the last century.

